# Demographics, longitudinal changes and outcome of high blood pressure in children and adolescents on kidney replacement therapy: 15 years of data from the ESPN/ERA Registry

**DOI:** 10.1007/s00467-026-07219-4

**Published:** 2026-03-25

**Authors:** Enrico Vidal, Jérôme Harambat, Sevcan A. Bakkaloglu, Manish D. Sinha, Cornel Aldea, Charalampos Kapogiannis, Kajsa Åsling Monemi, Sergey Baiko, Roberto Chimenz, Dorota Drozdz, Thomas Giner, James G. Heaf, Dmytro Ivanov, Augustina Jankauskiene, Regula Laux-End, Adrian Lungu, Anette Melk, Christine Pietrement, David Pitcher, Ludmila Podracka, Tomas Seeman, Carla Simão, Yılmaz Tabel, Juuso Tainio, Dean Wallace, Kitty J. Jager, Marjolein Bonthuis

**Affiliations:** 1 Pediatric Nephrology Unit, Department of Woman’s and Child’s Health, University Hospital of Padua, Padua, Italy; 2 Department of Medicine (DMED), University of Udine, Udine, Italy; 3 Pediatric Nephrology Unit, Bordeaux University Hospital, Bordeaux, France; 4 Department of Pediatric Nephrology, Gazi University Faculty of Medicine, Ankara, Türkiye; 5 Department of Paediatric Nephrology, King’s College London, Evelina London Children’s Hospital, London, UK; 6 Pediatric Nephrology Department, Emergency Clinical Hospital for Children, Cluj-Napoca, Romania; 7 Department of Pediatric Nephrology, Agia Sophia Children’s Hospital, Athens University, Athens, Greece; 8 Paediatric Department, Athens Medical Group, Athens, Greece; 9 Department of Clinical Science, Intervention and Technology, Division of Paediatrics, Karolinska Institutet, Karolinska University Hospital Huddinge, Stockholm, Sweden; 10 Department of Pediatrics, Belarusian State Medical University, Minsk, Belarus; 11 Pediatric Nephrology and Dialysis Unit, University Hospital “G. Martino”, Messina, Italy; 12 Department of Pediatric Nephrology and Hypertension, Pediatric Institute, Jagiellonian University Medical College, Cracow, Poland; 13 Department of Pediatrics I, Medical University of Innsbruck, Innsbruck, Austria; 14 Department of Medicine, Zealand University Hospital, Roskilde, Denmark; 15 Department of Nephrology and RRT, Shupyk, National Health Care University, Kyiv, Ukraine; 16 Pediatric Center, Institute of Clinical Medicine, Vilnius University, Vilnius, Lithuania; 17 Children’s Hospital St. Gallen, St. Gallen, Switzerland; 18 Pediatric Nephrology Department, Fundeni Clinical Institute, Bucharest, Romania; 19 Department of Pediatric Kidney, Liver and Metabolic Diseases, Hannover Medical School, Hannover, Germany; 20 Department of Pediatrics, Reims University Hospital, Reims, France; 21 UK Renal Registry, The Renal Association, Bristol, UK; 22 Department of Pediatrics, Medical Faculty, Comenius University, Bratislava, Slovakia; 23 Department of Pediatrics, 2nd Medical Faculty, Charles University Prague and University Hospital Ostrava, Ostrava, Czech Republic; 24 Department of Pediatrics, Hospital de Santa Maria, CHULN, Lisbon Faculty of Medicine, FMUL, Lisbon, Portugal; 25 Department of Pediatric Nephrology, Faculty of Medicine, İnönü University, Malatya, Türkiye; 26 Department of Pediatric Nephrology and Transplantation, New Children’s Hospital, University of Helsinki and Helsinki University Hospital, Helsinki, Finland; 27 Department of Paediatric Nephrology, Royal Manchester Children’s Hospital, Manchester, UK; 28 ESPN/ERA Registry, Department of Medical Informatics, Amsterdam UMC location University of Amsterdam, Amsterdam, the Netherlands; 29 Amsterdam Public Health, Quality of Care, Amsterdam, the Netherlands

**Keywords:** Pediatrics, Dialysis, Transplantation, Hypertension, Antihypertensive medications

## Abstract

**Background:**

Hypertension (HTN) is a well-known complication among patients on pediatric kidney replacement therapy (KRT). We aimed to evaluate demographics, longitudinal changes and outcomes of high blood pressure (BP) among children and adolescents on KRT.

**Methods:**

Data on BP and antihypertensive (AH) medications reported to the ESPN/ERA Registry on 6071 patients from 28 European countries starting KRT < 20 years of age between 2007 and 2021, were included.

**Results:**

Hypertension (HTN), AH medication use, and uncontrolled HTN were reported in 60.7%, 45.0%, and 32.0% of patients, respectively. Prevalence of uncontrolled HTN was 49.7% in HD, 42.3% in PD, and 27.3% in transplanted patients. Younger age, dialysis, and shorter KRT vintage were risk factors for uncontrolled HTN. AH medication use was lower among young patients, females and those on dialysis, and higher with a shorter KRT vintage and non-CAKUT kidney disease. Among AH medication users, 27.9% of transplantation, 48.1% of PD and 58.9% of HD patients showed a systolic BP > 95th percentile. Uncontrolled HTN significantly decreased over time in HD patients (52.3% at dialysis start vs. 42.5% after 5 years; annual percentage change [APC] − 3.5%; 95%CI: − 6.2; -0.7), despite similar AH medication use. After 5 years, transplanted patients showed a significant reduction in both prevalence of uncontrolled HTN (APC − 3.6%; 95%CI: − 5.7; − 1.5) and AH medication use (APC − 1.6%; 95%CI: − 2.6; − 0.6%). No trends were found for PD patients. Uncontrolled HTN was not associated with mortality (aHR 1.02; 95%CI: 0.79–1.33).

**Conclusions:**

HTN is highly prevalent in children and adolescents on KRT. Younger children and HD patients should be carefully evaluated for BP status after entering dialysis or shortly after transplantation.

**Graphical abstract:**

**Figure:**
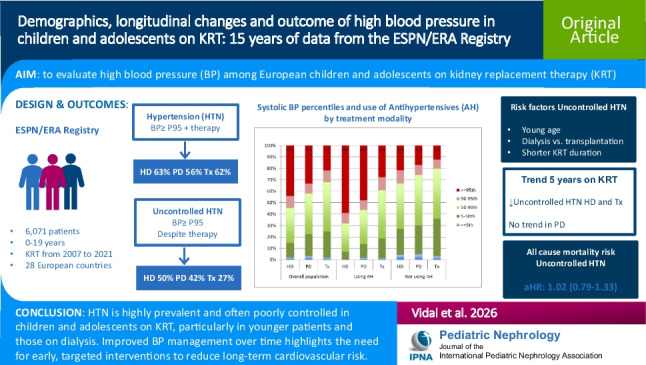
A higher resolution version of the Graphical abstract is available as Supplementary information

**Supplementary Information:**

The online version contains supplementary material available at 10.1007/s00467-026-07219-4.

## Introduction

The appropriate management of blood pressure (BP) is mandatory for the prevention of short- and long-term adverse outcomes in children receiving kidney replacement therapy (KRT). Children constitute a unique patient group in whom control of cardiovascular risk factors are key to improving long-term survival. Still, the prevalence of cardiovascular risk factors in pediatric KRT remains very high, and cardiovascular disease is a major cause of death [1–4]. Hypertension (HTN) represents the most common modifiable risk factor for the occurrence of cardiovascular disease in children on dialysis, being present in about 55% to 80% of patients receiving KRT [5–11]. In contrast to numerous studies in adults, little has been reported on longitudinal BP patterns in the pediatric KRT population in Europe. Furthermore, existing information is usually limited to HTN in dialysis patients, whereas information on goals of HTN management and data on the clinical course of BP in transplanted patients are scarce [12–15].

In a previous ESPN/ERA Registry study, prevalence of and risk factors for HTN among 3337 pediatric KRT patients from 15 European countries were reported [8]. Uncontrolled HTN was significantly more prevalent in very young patients, during the first year of KRT, and higher in those on hemodialysis (HD) compared to kidney transplant recipients and those on peritoneal dialysis (PD).

In the present study, we aimed to further expand the analyses in this population by reporting the clinical course of BP during follow-up in children and adolescents on KRT over a larger population and over an extended and more recent time period. The ESPN/ERA Registry provides a unique opportunity to study BP over time, now with data collected over 15 years, in a Europe-wide setting. In detail, we aimed to determine (1) the BP distribution, the prevalence of high BP and its 15-year clinical course in the European pediatric and adolescent KRT population, (2) the use of antihypertensive (AH) medication and its association with BP values over time, and to evaluate (3) the association between BP levels and mortality.

## Methods

### Subjects and definitions of variables

Patient data were extracted from the ESPN/ERA Registry, a population-based registry that collects data on pediatric and adolescent KRT patients in Europe on an annual basis. The Registry comprises two datasets. Core or essential data are provided for all patients, whereas extended data, including clinical variables, are collected on a voluntary basis and are thus not available for every patient. All patients who started KRT before the age of 20 years, between 2007 and 2021, from 28 European countries providing data on BP and AH medication were included. Countries were grouped according to their gross domestic product per capita (GDP) quartiles: Moldova, Georgia, Albania, Serbia, and North Macedonia (first quartile), Belarus, Bulgaria, Croatia, Türkiye, Latvia, Greece, and Hungary (second quartile), Poland, Slovakia, Portugal, Estonia, Lithuania, Slovenia, Czech Republic, and Spain (third quartile), Italy, Malta, Belgium, Ireland, Germany, the Netherlands, Norway and Switzerland (fourth quartile). The Medical Ethics Review Committee of the Amsterdam Medical Center, the Netherlands provided a waiver for ethical approval of this study (W21_257# 21.283).

For dialysis patients, BP was measured before a dialysis session. Systolic and diastolic BP standard deviation scores (SDS) were calculated for each subject according to age, sex and height using the 2016 European Society of Hypertension guidelines on high blood pressure in children and adolescents [16]. HTN was defined as either office systolic BP (SBP) or diastolic BP (DBP) ≥ 95th percentile (SDS ≥ 1.65), or when using AH medications. Uncontrolled HTN was defined as a systolic or diastolic BP SDS ≥ 95th percentile, irrespective of AH medication use. The primary kidney disease was classified according to pre-existing European Renal Association (ERA) groups adapted for children and causes of death were defined by the ERA coding system [17].

### Statistical analyses

Multivariable generalized linear models and linear mixed models were used to provide estimates using longitudinal data and to evaluate BP values over time for each KRT modality (HD, PD and kidney transplantation), adjusting for the following variables: age, sex, primary kidney disease, country GDP, and interaction between time and dialysis modality, whenever appropriate according to the criteria for confounding [18].

To examine the prevalence of HTN and use of AH medication over time, Joinpoint regression software provided by the Surveillance Research Program of the US National Cancer Institute was used [19]. Joinpoint regression allows the identification of time points where a significant change in the linear slope of a trend occurs. The annual percentage change (APC) was computed using Poisson regression provided by the Joinpoint regression software [20].

Time-varying Cox regression was used to evaluate the association between BP status and all-cause mortality. Adjustments were made for the confounding effects of age, sex, country GDP, primary kidney disease, and KRT modality.

Information about AH medication use was unavailable in 39.7% of HD, 49.8% of PD, and 13.4% of transplanted patients. The impact of missing data was checked in sensitivity analyses using multiple imputation with five imputed datasets.

Analyses were performed using SAS 9.4 statistical software package (SAS Institute, Cary, NC, USA) and JoinPoint version 4.7.0.0 [19].

## Results

### Baseline characteristics

We included 6071 patients contributing a total of 35,663 measurements. At time of first BP measurement, the median age of patients was 11.8 (IQR: 6.6–15.5) years, 59% were male, and CAKUT was the most common cause of kidney disease (33%). After a median (IQR) time on KRT of 0.9 (0.3–3.1) years, 21% of children were on HD, 30% on PD and 49% had a functioning kidney graft. Patient characteristics are detailed in Table 1. Furthermore, 82% of HD patients were older than 7 years, while 55.4% of PD patients were older than 7 years of age. A small minority (8%) of kidney transplant recipients were younger than 4 years of age, while most of the transplanted patients were adolescents (49% ≥ 13 years).

**Table 1:** Demographic and clinical characteristics of study population at time of first BP measurement after start of KRT

Variable	All (*n* = 6071)
Sex:	
- Male	3584 (59%)
- Female	2487 (41%)
Age (years):	
- Median (IQR)	11.8 (6.6–15.5)
- 0–3	955 (15.7%)
- 4–6	664 (10.9%)
- 7–12	1832 (30.2%)
- 13–16	1830 (30.1%)
- ≥ 17	790 (13.1%)
Primary kidney disease:	
- Glomerulonephritis	1130 (18.6%)
- CAKUT	1995 (32.9%)
- Cystic kidneys	814 (13.4%)
- Hereditary nephropathy	288 (4.7%)
- Ischemic renal failure	106 (1.8%)
- HUS	234 (3.9%)
- Metabolic disorders	198 (3.3%)
- Vasculitis	81 (1.3%)
- Miscellaneous	732 (12.1%)
- Unknown/missing	493 (8.1%)
Height SDS (median, IQR) (available for 5964 cases)	− 1.68 (− 2.78; − 0.73)
Weight SDS (median, IQR) (available for 6058 cases)	− 0.87 (− 2.05; + 0.06)
BMI SDS (available for 5578 cases)	
- Median (IQR)	− 0.02 (− 0.98; +0.80)
- ≤ 5th percentile (≤ −1.64 SDS)	742 (13.3%)
- 6th–84th percentile (> −1.64 to 1.04 SDS)	3757 (67.4%)
- ≥ 85th percentile (≥ 1.04)	1079 (19.3%)
Comorbidity (available for 4722 cases):	
- Yes	2573 (54.5%)
- No	2149 (45.5%)
KRT modality:	
- HD	1301 (21.4%)
- PD	1789 (29.5%)
- Tx	2981 (49.1%)
Use of AH medications (available for 3584 cases):	
- Yes	2593 (61.6%)
- No	1617 (38.4%)

Abbreviations: *IQR*, interquartile range; *CAKUT*, congenital anomalies of the kidney and urinary tract; *HUS*, hemolytic-uremic syndrome; *SDS*, standard deviation score; *BMI*, body mass index; *KRT*, kidney replacement therapy; *HD*, hemodialysis; *PD*, peritoneal dialysis; *Tx*, kidney transplantation; *AH*, antihypertensive medications

### Prevalence of hypertension and use of AH medication

In the overall cohort, mean (± standard error (SE)) SBP and DBP standard deviation scores (SDS) were 0.92 (± 0.01) and 0.74 (± 0.01), respectively. SBP and DBP SDS were higher in younger patients and in those on HD, as compared with PD or transplanted patients (Supplementary Fig. 1). Hypertension was present in 60.7% of patients, showing little variation across the treatment modalities: 63.0% in HD, 55.6% in PD, and 61.6% in transplanted patients. The prevalence of uncontrolled HTN was 32% in the overall cohort and 49.7% in HD, 42.3% in PD, and 27.3% in the transplanted patients.

**Fig. 1:**
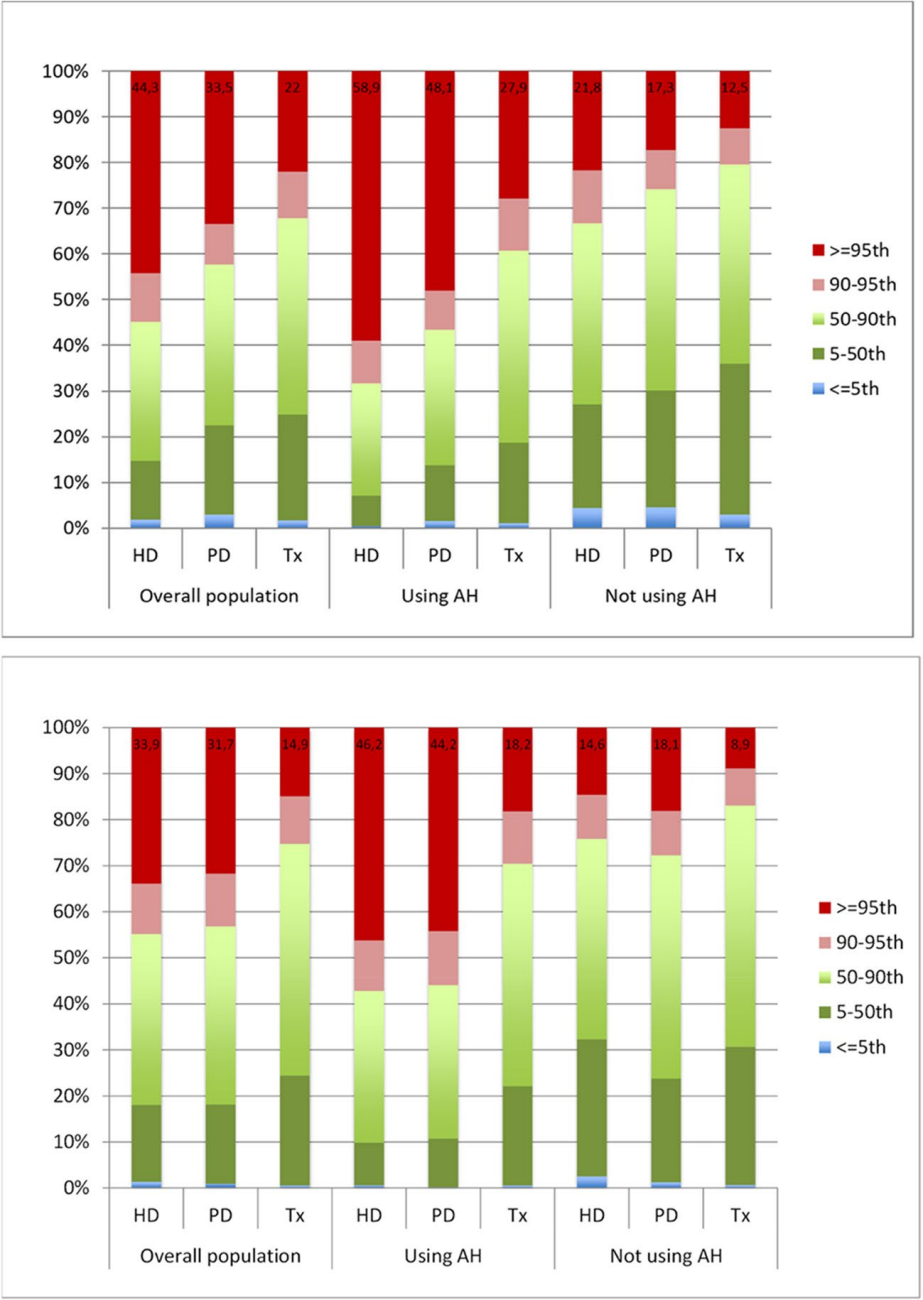
Distribution of systolic (upper panel) and diastolic (lower panel) BP percentiles according to AH medication use and type of KRT, Abbreviations: HD, hemodialysis; PD, peritoneal dialysis; Tx, kidney transplantation; AH, antihypertensive medications

Overall, 45% of patients were treated with AH medications. AH medication use was reported in 35.0% of patients younger than 3 years, in 41.3% of those 4–6 years old, in 43.5% of those 7–12 years old, in 46.4% of those 13–16 years old, and in 49.4% of those older than 17 years of age (*p* < 0.001). Transplant recipients (51.0%) received AH medications more frequently as compared to patients on HD (37.7%) and those on PD (30.0%). In AH medication users, 58.9% of HD patients, 48.1% of PD patients, and 27.9% of transplanted patients had a systolic BP above the 95th percentile. Among those not treated with AH medications, 21.8% of HD patients, 17.3% of PD patients, and 12.5% of transplant recipients had a systolic BP above the 95th percentile. Similar but slightly attenuated figures were observed for diastolic BP (Fig. 1).

### Longitudinal analyses

**Fig. 2:**
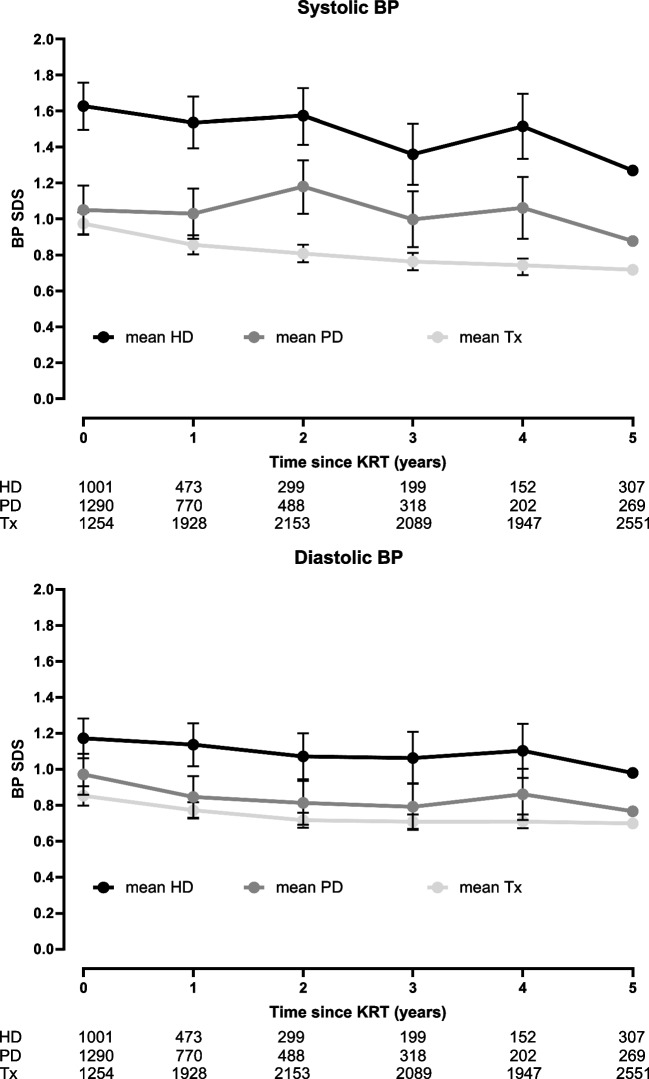
Mean systolic and diastolic BP SDS over time among children on KRT. Data are adjusted for sex, PRD, country GDP, age at time of measurement, and interaction between time and dialysis modality. Abbreviations: BP, blood pressure; SDS, standard deviation score; HD, hemodialysis; PD, peritoneal dialysis; Tx, kidney transplantation; KRT, kidney replacement therapy; PRD, primary renal disease; GDP, Gross Domestic Product

Mean (± SE) systolic BP at KRT initiation was 1.63 (± 0.07), 1.05 (± 0.07) and 0.97 (± 0.03) SDS in HD, PD and transplanted patients, respectively, while after 5 years it was 1.27 (± 0.05), 0.88 (± 0.05), and 0.72 (± 0.05) SDS in the corresponding KRT categories. Results of longitudinal analysis for both systolic and diastolic BP according to KRT modality over time are shown in Fig. 2.

**Fig. 3:**
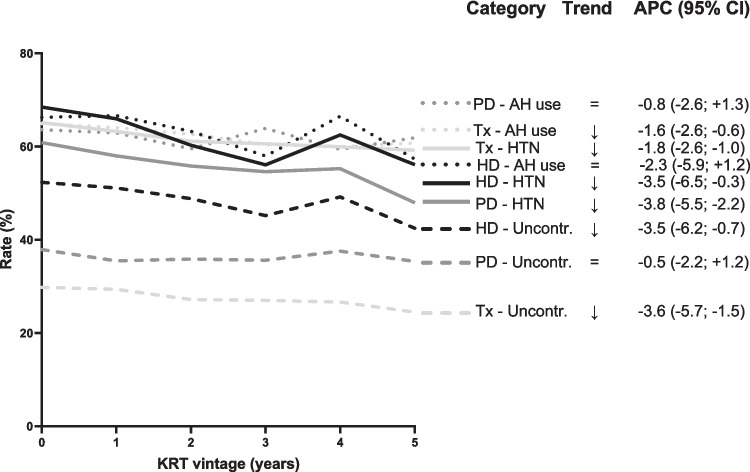
Prevalence of HTN and AH use according to KRT vintage. Rates are adjusted by age, sex, PRD, country GDP. Arrows indicate a statistically significant variation in the trend. Abbreviations: APC, annual percentage change; CI, confidence interval; PD, peritoneal dialysis; AH, antihypertensive medications; Tx, kidney transplantation; HTN, hypertension; HD, hemodialysis; PD, peritoneal dialysis; Uncontr., uncontrolled hypertension; KRT, kidney replacement therapy; PRD, primary renal disease; GDP, Gross Domestic Product

**Fig. 4:**
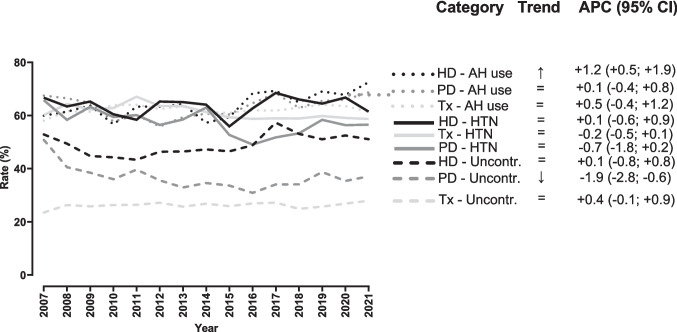
Prevalence of uncontrolled HTN and AH use according to calendar years. Rates are adjusted by age, sex, PRD, country GDP. Arrows indicate a statistically significant variation in the trend. Abbreviations: APC, annual percentage change; CI, confidence interval; HD, hemodialysis; AH, antihypertensive medications; PD, peritoneal dialysis; Tx, kidney transplantation; HTN, hypertension; Uncontr., uncontrolled hypertension; PRD, primary renal disease; GDP, Gross Domestic Product

During the KRT course, the risk of uncontrolled HTN was higher for young patients. Being on dialysis (HD vs. transplantation, OR 2.49 [95% CI: 2.23–2.77]; PD vs. transplantation, OR 1.47 [95% CI: 1.32–1.63]), and shorter KRT vintage (< 1 vs. > 5 years, OR 1.38 [95% CI: 1.27–1.51]), were also risk factors for uncontrolled HTN. The likelihood of using AH medication over time was significantly lower in younger children and in dialysis patients (compared with transplanted), while it was higher for female patients, those with a KRT vintage shorter than 3 years, and in patients with non-CAKUT kidney disease (Table 2). All estimates obtained using imputed values were consistent with those obtained using the original data. Prevalence of hypertension decreased after start of KRT for all treatment modalities. Patients on HD showed a significant decrease in the prevalence of uncontrolled HTN over time (52.3% at dialysis start vs. 42.5% after 5 years; annual percentage change [APC] − 3.5%; 95% CI: − 6.2; − 0.7; *p* < 0.05), despite similar AH medication use (APC − 2.3%; 95% CI: − 5.9; + 1.2). PD patients showed no reduction in prevalence of uncontrolled HTN (APC − 0.5%; 95% CI: − 2.2; + 1.2), or AH medication use over time (APC − 0.8%; 95% CI: − 2.6; + 1.3). After 5 years, transplanted patients showed a reduction in both prevalence of uncontrolled HTN (APC − 3.6%; 95% CI: − 5.7; − 1.5; *p* < 0.05) and use of AH medications (APC − 1.6%; 95% CI: − 2.6; *-*0.6%; *p* < 0.05) (Fig. 3). Prevalence of uncontrolled HTN according to calendar years remained stable in HD and kidney transplant patients, whereas in patients on PD it decreased from 50.8% in 2007 to 37.1% in 2021, with − 1.9% per year (95% CI: − 2.8; − 0.6; *p* < 0.05). The APC for the use of AH medication increased with calendar year in HD (+ 1.2%; 95%CI: + 0.5; + 1.9), but remained stable for PD and transplanted patients (Fig. 4).

**Table 2:** Likelihood of uncontrolled hypertension and use of antihypertensive medications over time (obtained by generalized linear models)

Parameter	Uncontrolled hypertension		Use of antyhypertensive medications	
	OR	95% CI	OR	95% CI
Sex^a^:				
• Male	Ref		Ref	
• Female	1.05	0.98–1.13	0.86	0.78–0.95
Age group^b^:				
• 0–3 years	3.94	3.50–4.43	0.59	0.52–0.67
• 4–6 years	2.24	2.00–2.50	0.74	0.67–0.83
• 7–12 years	1.52	1.39–1.66	0.83	0.77–0.90
• 13–16 years	1.16	1.07–1.25	0.93	0.88–0.99
• ≥ 17 years	Ref		Ref	
KRT modality^c^:				
• HD	2.49	2.23–2.77	0.70	0.62–0.79
• PD	1.47	1.32–1.63	0.61	0.55–0.68
• Transplantation	Ref		Ref	
KRT duration^d^:				
• 0–0.99 years	1.38	1.27–1.51	1.14	1.04–1.24
• 1–1.99 years	1.10	1.01–1.21	1.14	1.05–1.24
• 2–2.99 years	0.94	0.86–1.02	1.14	1.05–1.23
• 3–3.99 years	0.89	0.81–0.97	1.06	0.99–1.14
• 4–4.99 years	0.92	0.84–0.99	1.03	0.97–1.09
• ≥ 5 years	Ref		Ref	
Primary kidney disease^e^:				
• CAKUT	Ref		Ref	
• Glomerulonephritis	1.06	0.96–1.17	1.98	1.75–2.24
• Cystic disease	0.88	0.79–0.98	1.56	1.36–1.80
• Hereditary	0.98	0.79–1.20	1.59	1.27–1.98
• Ischemic renal failure	0.93	0.72–1.20	1.42	1.04–1.95
• HUS	0.93	0.77–1.21	2.34	1.82–3.01
• Metabolic	1.03	0.83–1.26	1.63	1.28–2.08
• Vasculitis	1.02	0.74–1.41	3.97	2.71–5.82
• Miscellaneous	0.84	0.74–0.94	1.03	0.89–1.20
• Unknown/missing	0.84	0.73–0.97	1.62	1.37–1.93

^a^ Adjusted for country GDP

^b^ Adjusted for sex, primary kidney disease, and country GDP

^c^ Adjusted for sex, primary kidney disease, age, KRT duration, and country GDP

^d^ Adjusted for sex, primary kidney disease, age, and country GDP

^e^ Adjusted for sex, age, and country GDP

Abbreviations: *OR*, odds ratio; *CI*, confidence interval; *KRT*, kidney replacement therapy; *HD*, hemodialysis; *PD*, peritoneal dialysis; *CAKUT*, congenital anomalies of the kidney and urinary tract; *HUS*, hemolytic-uremic syndrome; *GDP*, Gross Domestic Product

### Association of HTN with mortality

After adjustment for confounding factors, uncontrolled HTN and BP SDS were not associated with increased all-cause mortality risk in time-varying Cox regression models: uncontrolled HTN (aHR: 1.02, 95% CI: 0.79–1.33), systolic (aHR: 1.02, 95% CI: 0.94–1.12), and diastolic BP SDS (aHR: 0.96, 95% CI: 0.86–1.07) (Supplementary Table 1).

## Discussion

Our study further confirms that HTN is highly prevalent among children and adolescents on KRT. In European dialysis patients, 50% of children on HD and 42% of those on PD had a BP ≥ 95th percentile, irrespective of medication use. When the use of AH medications was included in the definition of HTN, the prevalence of HTN rose to 63% in children on HD and 56% in those on PD. In comparison, in transplanted patients, prevalence of uncontrolled HTN and HTN overall (uncontrolled + use of AH medications) was 27% and 62%, respectively.

Our current findings align with previously reported prevalences of HTN in pediatric dialysis patients ranging from 55% in a recent analysis from the International Pediatric Hemodialysis Network [11] to 69% in a previous ESPN/ERA Registry study including patients enrolled in the Registry from 1999 to 2010 [8] and 78% in US pediatric dialysis patients [5, 7]. With a current prevalence of 62% among pediatric and adolescent transplant recipients, HTN seemed to be more common than reported previously in European (50%) [8] and similar to US patients (58%) [12]. In addition, a recent study from Khandelwal et al. reported that HTN is already very common in children approaching KRT [21]. In this study, the authors combined data on 248 pediatric patients with stages 4–5 CKD approaching KRT from two prospective multicenter cohorts in Europe and Canada and found a high prevalence of modifiable cardiovascular risk factors, including HTN in 51% of patients.

The persistence of high prevalence of HTN in this high-risk population needs further consideration. Currently, there are no data comparing specific BP targets in children on dialysis. Most experts would agree that HTN should be treated in this population with BP levels targeted < 90th percentile. In those following kidney transplantation, lower BP targets have been suggested although robust data are lacking. It is likely that lack of specific targets for children and adolescents on KRT result in sub-optimal achievement of BP control in this population.

Despite these issues, our study allows the identification of patients at risk of hypertension after KRT: the youngest children and HD patients should be particularly carefully evaluated for BP status following initiation of dialysis or soon after transplantation. Being on dialysis is a risk factor for HTN as compared with transplantation, and younger age likely is a barrier for an efficient use of AH medications. Several factors might impact the inadequate BP control in small children, including the low utilization of AH therapy possibly due to a lack of pediatric labelling data for many medications, especially combination drugs, and limitations in BP assessment due to the lack of a full range of cuff sizes in some countries. Small patient size often prevents the use of ambulatory BP monitoring (ABPM), because of the lack of reference values for children < 5 years of age or 120 cm in height, whereas the BP status defined using casual measurements may be inaccurate especially in younger children [22]. In this setting, under-diagnosis of HTN could also explain the lack of AH treatment in the youngest patients, resulting in a higher prevalence of untreated HTN.

Sustained control of HTN over time is perhaps the most important factor in reducing future cardiovascular risk [23], but few data are available on whether this can be achieved in pediatric patients with kidney failure [24]. In our study, the longitudinal analysis showed that a more adequate control of BP over time is obtained in children and adolescents on KRT. As compared with PD and transplanted patients, children on HD still exhibit higher mean systolic and diastolic BP levels over time. However, there is a progressive reduction in the prevalence of uncontrolled HTN despite similar use of AH medications. In contrast the prevalence of uncontrolled HTN in PD patients did not change over time. These findings probably reflect a different evolution in fluid management and volume overload in children on long-term dialysis. We postulate that control and optimization of fluid volume were probably improved with HD vintage, as a result of a cardiovascular adaptation, better assessment of dry weight, and/or use of intensified dialysis regimens [25–27]. In contrast, fluid overload tends to get worse in children on PD due to a progressive decrease in residual kidney function [28].

In our cohort (uncontrolled) HTN decreased after kidney transplantation together with a simultaneous decrease in the use of AH medications. In a retrospective analysis of 336 pediatric kidney transplant recipients from the Cooperative European Pediatric Renal Transplant Initiative (CERTAIN) Registry, 84% of children were hypertensive at discharge after successful transplantation, with 46% showing uncontrolled HTN. A higher systolic BP SDS was associated with shorter time since transplantation, whereas the proportion of normotensive and controlled hypertensive children increased from time at discharge to 1 year post-transplant [29]. In addition to younger age, high calcineurin inhibitors exposure, and a shorter time since transplantation were other determinants of a higher BP [29]. Hypertensinogenic effects of calcineurin inhibitors are dose-related, thus are prominent in the early post-transplant period, and depend on effects on vascular tone, sympathetic nervous system and kidney sodium retention [30]. Lower BP levels in those following kidney transplantation have also been shown to be associated with slower rates of progression from non-hypertensive to hypertension [31].

Sex is known to affect BP from the onset of puberty onwards [32] though underlying pathophysiologic mechanisms are poorly defined. However, many hypotheses have been proposed, with the hormonal component being prevailing [33]. In pediatric kidney transplant recipients, male sex was associated with higher BP values, even at younger age [29]. In our study, sex was not associated with a different risk of uncontrolled HTN, but females were less likely to use AH medications.

Overall, 45% of patients were treated with AH medications and transplant recipients received AH medications more frequently than patients on dialysis. However, even among treated patients, systolic BP was poorly controlled in half of the dialysis patients and nearly one-third of the transplanted patients. In the adult population, medication-related costs were a potential barrier for effective treatment of HTN [34]. The increasing prevalence of HTN and the continuous increasing expense of its treatment may influence prescribing patterns among physicians and treatment compliance by patients [35]. In fact, median drug costs for monotherapies involving diuretics, beta blockers and calcium channel blockers are substantially lower than those involving ACE inhibitors or combinations that are not labeled for use in children [34].

In our study, BP levels and status were not associated with mortality in children on KRT. Nevertheless, in a retrospective study with data from the US Renal Data Systems (USRDS), 23% of deaths from 1990 to 1996 among patients who started KRT in childhood and died before 30 years of age were of cardiovascular origin [36]. Furthermore, in the Long-term Effects of Renal replacement therapy in Children (LERIC) study [37] among adult patients with KRT onset below < 15 years of age between 1972 and 1992 in the Netherlands, cardiovascular disease was the main cause of death during 1972–2000 (41%) and infections during 2000–2010 (32%), and the reduced burden of cardiovascular deaths in the more recent period was associated with a stricter cardioprotective management, that significantly reduced the risk for left ventricular hypertrophy, hypercholesterolemia and hypertension. Mortality in children and adolescents on KRT is relatively rare and as such probably does not represent an adequate short-term outcome measure in hypertensive children. Rather, the impact of HTN in children on KRT should be evaluated using measures of subclinical cardiovascular damage, including cardiac function and left ventricular geometry, and vascular stiffness [21, 38–40]. A recent study in pediatric patients with stages 4–5 CKD approaching KRT showed that subclinical cardiovascular damage already started in the years preceding KRT initiation, and modifiable cardiovascular disease risk factors, particularly diastolic BP and body mass index, were strongly associated with an increase of cardiovascular damage over time [21]. Moreover, studies in pediatric kidney transplant recipients indicate early cardiac and vascular alterations, such as left ventricular hypertrophy and subclinical myocardial dysfunction, which were associated with hypertension and kidney graft function. These findings further support the need for comprehensive cardiovascular monitoring in this vulnerable population [41, 42]. In addition, it is known that cardiovascular disease risk factors, such as HTN, track well into adulthood [43], indicating the need for long-term outcome studies and early diagnosis and timely management to protect for further cardiovascular damage in pediatric KRT patients.

Despite being based on a large pan-European cohort of children on KRT, our study has several limitations. It lacks some important information, such as pubertal stages, details on AH medication, type and dose of immunosuppressant drugs in transplanted patients. Data on BP and use of AH medications are collected on a voluntary basis which results in missing data. However, the results of the sensitivity analyses using imputed values were not different from those obtained using the original data. Moreover, there is limited information available on the method and frequency of BP measurement, and the goals of HTN management. ABPM is the reference standard for classification of HTN and accurate assessment of cardiovascular risk. In HD patients, ABPM is more informative than pre- and post-dialysis casual BP measurements and improves the predictability of BP as a risk factor for target organ damage. Diagnosis and treatment monitoring of HTN among pediatric dialysis patients is enhanced with addition of ABPM [44]. In a study from the Cardiovascular Comorbidity in Children with CKD (4C) Consortium, less than 50% of stage III–V CKD children with ambulatory HTN were identified by office BP, and almost 50% of the patients diagnosed as hypertensive in office were found normotensive by ABPM [45]. However, our study reflects clinical practice in most European countries, where casual office BP measurement is still the mainstay of BP evaluation and management in children on KRT.

In conclusion, HTN is highly prevalent and still inadequately treated in a large proportion of European children receiving KRT. Younger children and HD patients should be particularly carefully evaluated for BP status after entering dialysis or shortly after transplantation. Overall, in children and adolescents on KRT a more adequate control of BP is obtained over time.

## Supplementary Information

Below is the link to the electronic supplementary material.

Graphical abstract (PPTX 110 KB)

ESM 2 (DOCX 37.9 KB)

## Data Availability

The data underlying this manuscript cannot be shared with any third party because the regional/national registries that provided data to the ESPN/ERA Registry remain the owners of the data.
