# Supplementary material for: Demographics, longitudinal changes and outcome of high blood pressure in children and adolescents on kidney replacement therapy: 15 years of data from the ESPN/ERA Registry
Source: Pediatr Nephrol. 2026 Mar 25;41(9):2911–22. doi: 10.1007/s00467-026-07219-4 (PMC13423910; doi:10.1007/s00467-026-07219-4)
Supplement: Supplementary file 1 — Graphical abstract (PPTX 110 KB) [file 467_2026_7219_MOESM1_ESM.pptx]

## Slide 1
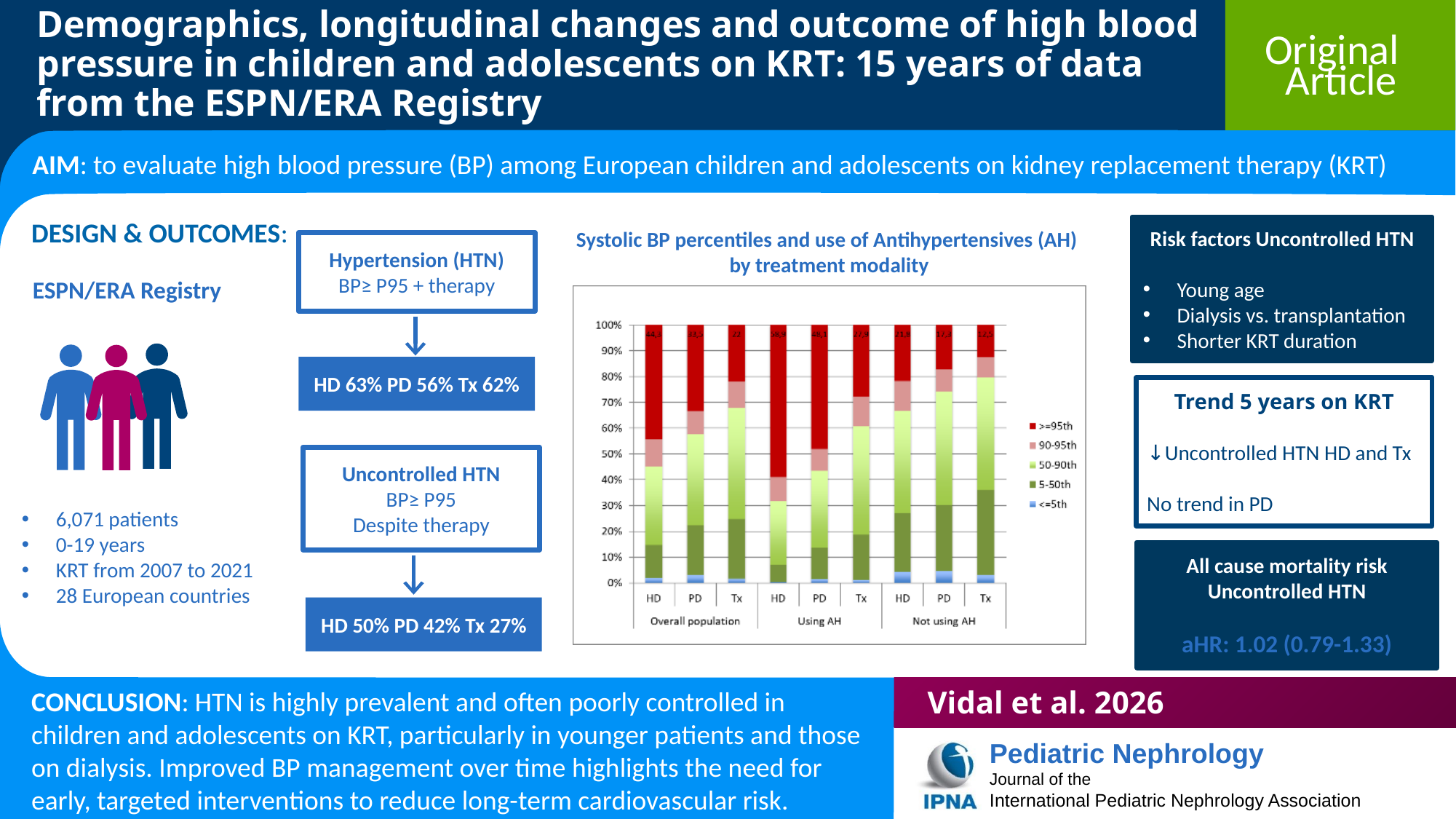

Demographics, longitudinal changes and outcome of high blood pressure in children and adolescents on KRT: 15 years of data from the ESPN/ERA Registry
AIM: to evaluate high blood pressure (BP) among European children and adolescents on kidney replacement therapy (KRT)
DESIGN & OUTCOMES:
Risk factors Uncontrolled HTN
Young age
Dialysis vs. transplantation
Shorter KRT duration
Systolic BP percentiles and use of Antihypertensives (AH)
by treatment modality
Hypertension (HTN)
BP≥ P95 + therapy
ESPN/ERA Registry
HD 63% PD 56% Tx 62%
Trend 5 years on KRT
↓Uncontrolled HTN HD and Tx
No trend in PD
Uncontrolled HTN
BP≥ P95
Despite therapy
6,071 patients
0-19 years
KRT from 2007 to 2021
28 European countries
All cause mortality risk
Uncontrolled HTN
aHR: 1.02 (0.79-1.33)
HD 50% PD 42% Tx 27%
Vidal et al. 2026
CONCLUSION: HTN is highly prevalent and often poorly controlled in children and adolescents on KRT, particularly in younger patients and those on dialysis. Improved BP management over time highlights the need for early, targeted interventions to reduce long-term cardiovascular risk.
