# Supplementary material for: Demographics, longitudinal changes and outcome of high blood pressure in children and adolescents on kidney replacement therapy: 15 years of data from the ESPN/ERA Registry
Source: Pediatr Nephrol. 2026 Mar 25;41(9):2911–22. doi: 10.1007/s00467-026-07219-4 (PMC13423910; doi:10.1007/s00467-026-07219-4)
Supplement: Supplementary file 2 — (DOCX 37.9 KB) [file 467_2026_7219_MOESM2_ESM.docx]

**Supplementary information**

**Supplementary Table 1. Unadjusted and adjusted association between BP status and mortality**

| **Variable** | **Unadjusted**  **HR (95% CI)** | **Adjusted^1^**  **HR (95% CI)** |
| --- | --- | --- |
| Systolic BP SDS | 1.14 (1.04–1.24) | 1.02 (0.94–1.12) |
| Diastolic BP SDS | 1.03 (0.97–1.10) | 0.96 (0.86–1.07) |
| Uncontrolled HTN | 1.30 (1.01–1.68) | 1.02 (0.79–1.33) |

^1^Adjusted for age, sex, primary kidney disease, country GDP, treatment modality

Abbreviations: BP, blood pressure; HR, hazard ratio; CI, confidence interval; SDS, standard deviation score; HTN, hypertension; GDP, Gross Domestic Product

**Supplementary Figure 1. Unadjusted standard deviation score (SDS) of systolic (A) and diastolic (B) blood pressure by treatment modality and age groups.** Box plot show the median, the 25^th^ and 75^th^ percentile and the 5^th^ and 95^th^ percentile.

Abbreviations: HD, haemodialysis; PD, peritoneal dialysis; Tx, kidney transplantation
